# Lightweight, Anisotropic, Compressible, and Thermally-Insulating Wood Aerogels with Aligned Cellulose Fibers

**DOI:** 10.3390/polym12010165

**Published:** 2020-01-08

**Authors:** Hao Sun, Hongjie Bi, Xin Lin, Liping Cai, Min Xu

**Affiliations:** 1 Key Laboratory of Bio-Based Material Science and Technology (Ministry of education), College of Material Science and Engineering, Northeast Forestry University, Hexing Road 26, Harbin 150040, China; sunhao@nefu.edu.cn (H.S.); bihongjie1016@163.com (H.B.); linxin7716@163.com (X.L.); 2 Mechanical and Energy Engineer Department, University of North Texas, Demon, TX 76201, USA; liping.cai@unt.edu; 3 College of Materials Science and Engineering, Nanjing Forestry University, Nanjing 210037, China

**Keywords:** wood aerogel, cellulose, multilayer structure, compressible, thermal insulation

## Abstract

The growing demand for lightweight, renewable, and excellent thermal insulation materials has fueled a search for high performance biomass materials with good mechanical compressibility and ultralow thermal conductivity. We propose a fabrication method for making a lightweight, anisotropic, and compressible wood aerogel with aligned cellulose fibers by a simple chemical treatment. The wood aerogel was mainly composed of highly aligned cellulose fibers with a relative crystallinity of 77.1%. The aerogel exhibits a low density of 32.18 mg/cm^3^ and a high specific surface area of 31.68 m^2^/g due to the removal of lignin and hemicellulose from the wood. Moreover, the multilayer structure of the aerogel was formed under the restriction of wood rays. Combined with a nanoscale pore, the aerogel presents good compressibility and an ultralow thermal conductivity of 0.033 W/mK. These results show that the wood aerogel is a high quality biomass material with a potential function of thermal insulation through optimizing structures.

## 1. Introduction

Energy consumption has become a major concern with regard to daily activity and industrial production, and has posed a huge burden on the environment and economy due to greenhouse gas emission and fossil fuel combustion [1,2]. Materials featuring low density, cost efficiency and high thermal insulation performance are highly desired for many applications in electrical, optical and structural fields. Expanded polystyrene as an important commercial thermal insulation materials has been widely used in electrical, vehicle and building insulation due to their light weight, low cost, simple process technology, and high thermal insulation performance [3,4]. However, the expanded polystyrene exhibits poor fire resistance and poor biodegradability, and releases toxic gas after combustion, which greatly increases the risk of their applications. Therefore, a lightweight, environmentally friendly, and ultralow thermal conductivity material is very necessary for practical applications.

Aerogels are considered as an ideal candidate for thermal insulation because of their ultralow density, highly porous structure, and low thermal conductivity [5,6,7]. Generally, silica aerogels and ceramic aerogels are the traditional inorganic aerogels with low density and high porosity for thermal insulation [8,9]. However, the intrinsic fragility of aerogel seriously hindered their large-scale application, especially when the external force changed. Several methods have been developed to optimize the structure for improving the mechanical performance of inorganic aerogels. For example, Dou et al. synthesized a silica nanofibrous aerogel by loading SiO_2_ on SiO_2_ nanofibers and SiO_2_ nanoparticle aerogels [10]. The aerogel showed resilient compressibility. Liang et al. fabricated SiC aerogels by graphitization and carbothermal reduction at ultrahigh temperatures [11]. Compared with the C aerogel, the compressive strength of the SiC aerogel was improved from 0.045 to 2.12 MPa. Despite the tremendous efforts that have been dedicated to endow inorganic aerogel with a high mechanical performance, the challenges due to the use of toxic modifiers, complex fabricating processes and extreme manufacturing conditions still remain.

Bio-based aerogels are produced from natural plant resources and degrade in the natural environment. Bio-based materials include cellulose, starch, pectin, and their derivatives [12]. Among them, cellulose is the most abundant polysaccharide polymer, being directly extracted from renewable biomass resources (wood, bamboo, and straw) and possessing high mechanical strength, high specific surface area, abundance of functional groups, and good biodegradability [13,14,15,16]. Cellulose aerogel as a lightweight, high porosity, and low thermal conductivity material is obtained from cellulose aqueous suspension through a freeze-drying method [17,18]. Generally, the preparation of aerogels from biomass materials involves multiple processes, including chemical purification, high temperature steaming, mechanical shatter, and further shearing and impacting in a specific machine [19,20,21]. In the above-mentioned process, specific equipment, complicated extracted procures, and external chemicals are employed, posing a large obstacle for its practical application.

Commonly, hardwood is mainly composed of wood fiber, wood ray, vessel, and parenchyma [22,23]. Intercross axial wood fibers and radial wood rays partially reflect the anisotropy of wood, which form a natural hierarchical structure. Presently, natural wood with anisotropic structures has attracted much attention for optical, nanofluidic, and ion transportation. Qiu et al. reported an optically transparent wood by impregnating delignified wood with PMMA [24]. The transparent wood with an anisotropic structure exhibited high transparency and high haze. By further loading tin oxide nanoparticles, the composites showed excellent near-infrared and ultraviolet absorbency. Jia et al. fabricated an anisotropic microfluidic framework via a delignification process from natural wood [25]. The natural microchannels have an ability to transport liquid and solid particles. G. Chen et al. developed a highly conductive cationic wood membrane via chemical modification and densification [26]. The densification process reduced a diameter of lumens and macropores, resulting in nanochannels and nanopores for ion transpiration. It is worth noting that natural wood with intrinsic porous (vessel and pit) and hierarchical structures has exhibited excellent thermal insulation performance since ancient times. The process of exacting cellulose from the natural wood in order to prepare aerogel not only ignored the natural hierarchical structure of wood, but also caused natural resource waste and energy consumption.

**Figure 1:**
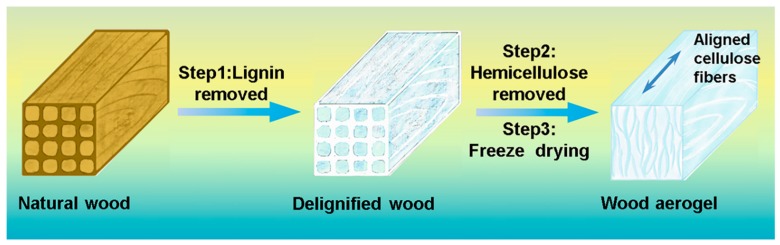
Schematic illustration of the fabricating of the wood aerogel.

In this study, wood aerogel with low density, high specific surface area, and mechanical compressibility for thermal insulation was fabricated via a facile process, as shown in Figure 1. The wood aerogel with a multilayer structure was obtained from the natural wood by removing lignin and hemicellulose in NaClO_2_/NaOH solution. The freeze-drying step was also critical to maintaining a multilayered and porous structure. The changes in appearance, microscopy morphology, and specific surface area of the treated specimens were evaluated by a setting time gradient. In addition, the thermal stability, mechanical compressibility, and thermal insulation performance of wood aerogel were further investigated.

## 2. Materials and Methods

### 2.1. Materials

Balsa wood (Ochroma pyramidale) specimens with a dimension of 20 mm × 20 mm × 10 mm (radial × tangential × longitudinal) were prepared. Sodium chlorite (NaClO_2_), acetic acid glacial (CH_3_COOH), and sodium hydroxide (NaOH) were purchased from Shanghai Aladdin Biochemical Technology Co., Ltd. (Shanghai, China). Distilled water was used in all experiments. Chemicals were analytical grade and used as received without further purification. The high surface emissivity 1712# tape was purchased from 3M.

### 2.2. Preparation of Wood Aerogels

The Balsa wood specimens were oven-dried at 103 °C for 24 h prior to the experiments. A solution of 2 wt % NaClO_2_ buffered with acetic acid at pH 4.6 was prepared for the delignification. The dried specimens were immersed in the prepared solution via the vacuum-pressure method to insure the specimens were fully impregnated with the solution. After being heated at 100 °C for 8 h, the specimens were washed with distilled water 3 times, and the delignified specimens were obtained. Then, the delignified specimens were immersed in a 5 wt % NaOH solution at 80 °C to remove hemicellulose in the wood. The alkali treatment time was 4 h, 8 h, and 12 h, respectively. The specimens were carefully washed with distilled water at 80 °C 3 times to remove the residual chemicals. Finally, the specimens were frozen in a freezer at −18 °C for 24 h and then dried in a freeze-dryer for 36 h to obtain the wood aerogel. The delignified specimens were frozen and dried as wood aerogel to obtain delignified wood.

### 2.3. Characterization

The morphology and structure of the wood specimens were characterized by scanning electron microscopy (SEM; Quanta 200, FEI, Hillsboro, OR, USA). X-ray diffraction (XRD) measurements were performed on an X-ray diffractometer (D/max 2200, Rigaku, Tokyo, Japan) in the range of 10°–80° with a scan rate of 4°/min. Fourier transform infrared spectroscopy (FTIR) was recorded on a spectrometer (Nicolet 6700, Thermo Scientific, Waltham, MA, USA) in the range of 600–4000 cm^−1^. Specific surface area and pore size distribution were calculated by the Brunauer–Emmett–Teller method on BET (JW-BK132F, JWGB, Beijing, China). Mechanical compressive tests of the wood specimens were conducted on a mechanical testing machine (AI-7000S TC160701511, Gotech, Taiwan, China) equipped with a 200 N load cell. The direction of the compressive force was perpendicular to the radial section. Thermal stability of the wood specimens was taken on the thermogravimetric analyzer (TGA; Q20, TA, Newcastle, DE, USA) from 30 to 800 °C with a heating rate of 10 °C/min in a nitrogen atmosphere. The thermal conductivity of wood specimens was determined by the transient hot-wire method using a thermal conductivity tester (TC3000, Xi’an Xiatech Electronic Technology Co., Ltd., Xi’an, China). Thermographic images of wood specimens were collected by an infrared thermal camera with a resolution of 320 × 240 pixels (E6, FLIR, Wilsonville, OR, USA). The α-cellulose, holocellulose, and lignin content were measured according to the GB/T744-1989, GB/T2677.10-1995, and GB/T2677.8-1994, respectively.

## 3. Results

### 3.1. Morphology and Structure

**Figure 2:**
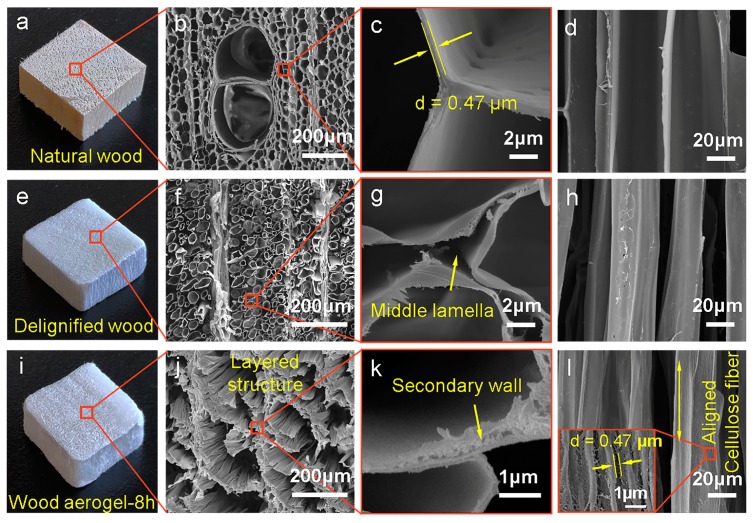
Morphology of different wood specimens. (**a**) Photograph of the natural wood. (**b**–**d**) scanning electron microscopy (SEM) images of natural wood. (**e**) Photograph of the delignified wood. (**f**–**h**) SEM images of delignified wood. (**i**) Photograph of the wood aerogel. (**j**–**l**) SEM images of wood aerogel. Inset of (l) showing a pore in the surface of cell wall.

The morphology and structures of wood specimens were characterized by SEM images. As shown in Figure 2a, the original balsa wood possessed a pale yellow appearance, simultaneously exhibiting a ring-shaped growth wheel in the cross section. In Figure 2b,c, the ordered pentagon cell lumen and elliptical vessels of the original balsa wood in cross section were observed. The thickness of the cell wall was only 0.47 μm and the ratio of the wall to the cavity was much smaller than 1, which demonstrated better performance in the elasticity of fibers [27]. In the tangential section of the wood, cells were closely connected and the cell surface was smooth without any wrinkles (Figure 2d). After the delignification, the color of the delignified wood turned to white, because most of lignin was oxidative-decomposed by acidified NaClO_2_ at 100 °C (Figure 2e). In Figure 2f,g, the middle lamella of the delignified wood was completely dissolved, resulting in the separation of wood cells. Due to the presence of a wood ray perpendicular to the wood fibers (Figure S1), the hierarchical structure of the wood aerogel was protected during the chemical treatment. In Figure 2h, wood fibers separated from each other were observed. Some small microfibrils were shedding from the surface of the cell wall, because lignin was also present on the primary and secondary walls of the cell. The wood aerogel was obtained from delignified wood by further removing hemicellulose. Interestingly, at visual inspection, the appearance of the natural wood was basically preserved in the wood aerogel (Figure 2i). However, the appearance of the wood aerogel was different from the natural wood and delignified wood in the low resolution SEM image. The regular cell structure was replaced with a multiply layered structure (Figure 2j). Moreover, a series of small damages occurred in the secondary wall and nanofiber was also observed in the high resolution image (Figure 2k), which was attributed to the hemicellulose degradation in the NaOH solution. In Figure 2l, cellulose fibers with good alignment on the cell surface were observed. Meanwhile, the nanoscale pore appearing in the inset of Figure 2l was related to the removal of lignin and hemicellulose.

### 3.2. Chemical Characterization

**Figure 3:**
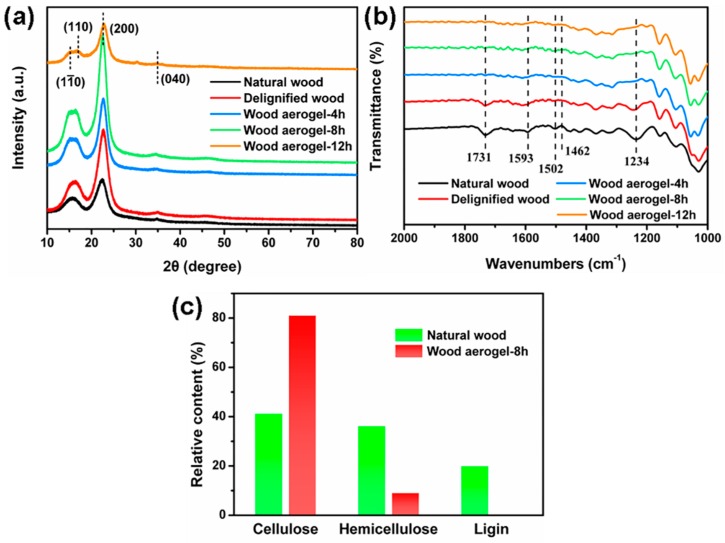
Chemical characterization of different wood specimens. (**a**) X-ray diffraction (XRD) patterns of different wood specimens. (**b**) Fourier transform infrared spectroscopy (FTIR) spectra of different wood specimens. (**c**) Relative content of cellulose, hemicellulose, and lignin of wood aerogel.

To further characterize the changes in the chemical structure of the wood specimens before and after the chemical treatment, XRD, FTIR, and a component content test were conducted. In Figure 3a, the diffraction peaks of the wood samples at around 14.9°, 16.5°, 22.7°, and 34.8° for (1$\bar{1}$0), (110), (200), and (040) planes are characteristic for cellulose I crystal, confirming that the types of cellulose crystal were not changed after chemical treatment [28,29]. The relative crystallinities of the samples were calculated according to the Segal peak height method [30]. It is worth noting that the relative crystallinity of the different wood specimens was changed periodically after the chemical treatment. The relative crystallinity of the natural wood, delignified wood, and wood aerogel were 62.3%, 75.9%, and 77.1%, respectively. This was because most amorphous regions of cellulose were removed at the initial stage of the chemical treatment. However, the relative crystallinity of specimens treated for 12 h slightly decreased to 68.8% because the peeling phenomena was conducted during the 12 h of processing [31]. The composition of the specimens was further determined by the infrared spectroscopy in Figure 3b. After the delignification, the peaks at 1593, 1502, and 1462 cm^−1^ belonging to aromatic skeletal vibrations of lignin disappeared, indicating lignin was removed. Moreover, after being treated with NaOH, the peaks at 1731 and 1234 cm^−1^ attributed to carbonyl stretching and C–O stretching also disappeared, demonstrating that hemicellulose was dissolved in the solution. To further demonstrate the above phenomena, the relative content of three components were tested in Figure 3c. The relative contents of cellulose, hemicellulose, and lignin of the natural wood were 41.38%, 36.38%, and 20.1%, respectively, while the relative contents of the wood aerogel were 81.28%, 9.25%, and 0.12%, respectively. This finding was consistent with that from the FTIR examination, further confirming the removal of lignin and hemicellulose from the natural wood.

### 3.3. Specific Surface Area and Pore Size Distribution Analysis

**Figure 4:**
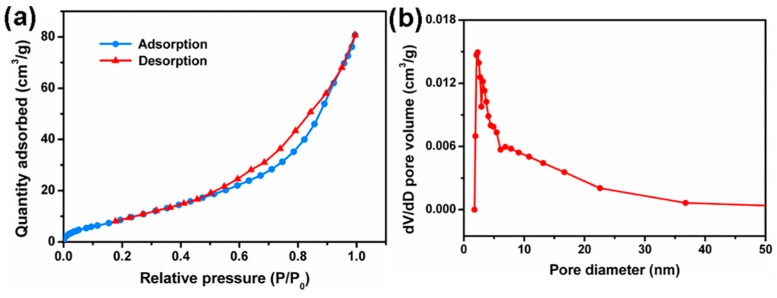
(**a**) Nitrogen adsorption–desorption isotherms of wood aerogel; (**b**) Mesopore size distribution of wood aerogel.

The specific surface area and mesopore size distribution of wood aerogel were characterized by the nitrogen adsorption-desorption isotherms and the Barrett–Joyner–Halenda (BJH) method, as shown in Figure 4. The wood aerogel exhibited type IV adsorption isotherms and possessed a type H3 hysteresis loop in Figure 4a [32,33]. The latter demonstrated the presence of a mesoporous structure in the wood aerogel. The pore diameters of the wood aerogel were mainly distributed at 2–40 nm (Figure 4b), which further confirmed the previous findings. In the process of removing lignin and hemicellulose, the middle lamella disappeared, exposing the nanoscale pores on the cell wall, resulting in a gradual increased in the specific surface area and pore volume of the wood specimens. The specific surface area and pore volume of the wood aerogel were 32.18 m^2^/g and 0.146 cm^3^/g, which were 4 times and 8 times higher than natural wood, respectively. However, the wood specimens treated for 12 h showed poor specific surface area and pore volume because of the collapse of the aerogel structure caused by the degradation of cellulose after the lengthy chemical treatment. Table 1 gives detailed information about the specific surface area, pore diameter, and pore volume of the wood specimens.

### 3.4. Thermal Stability

**Figure 5:**
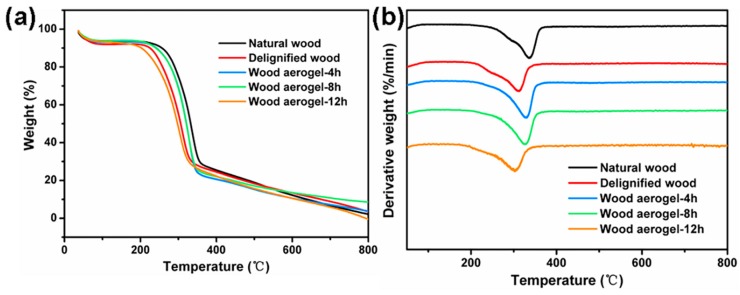
(**a**) Thermogravimetric analysis (TGA) curves and (**b**) Derivative Thermogravimetry (DTG) curves of different wood specimens.

The thermal stability of the wood aerogel was essential for thermal insulation applications. The TGA (thermogravimetric analysis) and DTG (Derivative Thermogravimetry) curves of the wood specimens are shown in Figure 5. Due to the inherent properties of biomass materials, all wood specimens began to degrade at 200 °C. Interestingly, the residue content of wood aerogel-8 h was higher than other specimens at 800 °C and the temperature of the maximum weight loss rate of the wood aerogel-8 h was 327 °C. The high thermal stability of the wood aerogel-8 h was attributed to its pure component (~81% cellulose) and high crystallinity (~77.1% relative crystallinity). However, the temperature of maximum weight loss rate of the natural wood was slightly higher than that of the wood aerogel-8 h, because the degradable temperature of lignin (160–900 °C) was higher than that of cellulose (315–400 °C) [34]. Furthermore, the specimens treated for 12 h showed the worst thermal stability, because the crystalline region of the cellulose was destroyed and the specific surface area was reduced. This result also revealed the effect of the structure on enhancing performance. By combining the specific surface area analysis, pore structure analysis, and thermal stability examination, the optimal treatment time (delignification process and sodium hydroxide treatment for 8 h) was further determined.

### 3.5. Mechanical Compressibility

**Figure 6:**
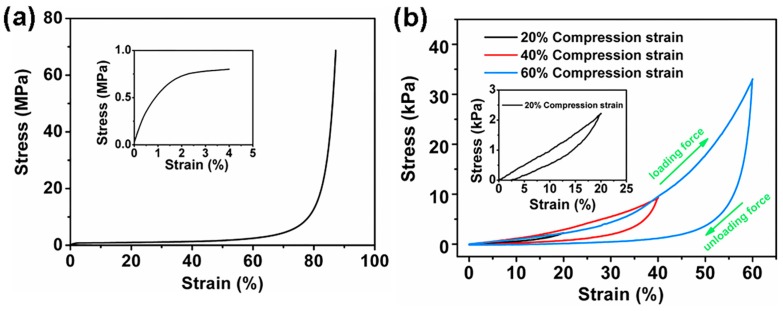
Stress-strain curves of the (**a**) natural wood and (**b**) wood aerogel-8 h under compression.

The mechanical compressibility of natural wood and wood aerogel-8 h was illustrated by stress-strain curves. In Figure 6a, the stress-strain curves show two distinct regions, including a linear elastic region at strain value <0.5% and a densification region at strain valve >0.5% [35]. In the first region, the compressive stress increased linearly with the strain, due to the bending of cell walls. In the densification region, the stress increased rapidly owing to the collapse and extrusion of cell. When external force was unloaded, the volume of the natural wood cannot be recovered. The stress-strain curves with different compression strain (20%, 40%, and 60%) for wood aerogel-8 h are shown in Figure 6b. There were also two distinct regions, including a linear elastic region below 20% compression strain and a densification region at strain value >20%. In the second region, the stress increased rapidly with the strain and the stress was 33.04 kPa at 60% compression value. Interesting, the strain can decrease to zero when the stress is unloaded, suggesting that the volume of wood aerogel-8 h can be recovered without deformation [36,37]. By removing the lignin and hemicellulose, the wood aerogel-8 h with layered structure was obtained, showing unique compressibility compared to natural wood.

### 3.6. Thermal Insulation

**Figure 7:**
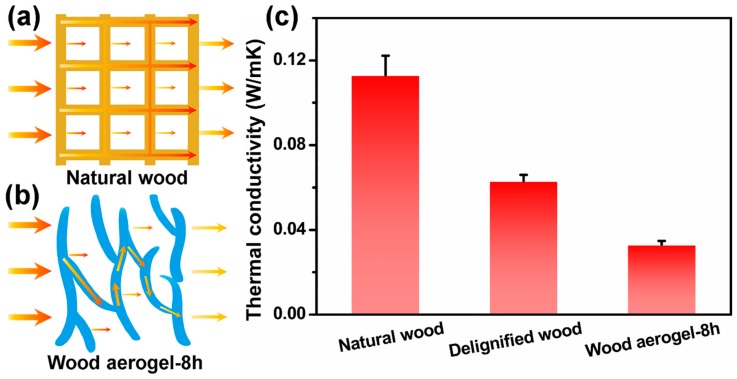
Thermal conductivity of wood specimens: (**a**) schematic of thermal conduction in natural wood; (**b**) schematic of thermal conduction in wood aerogel; (**c**) thermal conductivity of natural wood, delignified wood, and wood aerogel-8 h.

In addition to compression properties, thermal conductivity was also examined to evaluate the thermal insulation properties of wood aerogels. Figure 7a,b are schematics of heat conduction in the natural wood and wood aerogel-8 h, respectively. The thermal conductivity of the aerogel depends on the sum of heat conduction and heat radiation in the solid materials and gas phase [38,39]. The thermal conductivity factor, content, and density of the solid material change the thermal conductivity [40,41]. The main building blocks to assemble wood aerogel-8 h were purity and high crystalline cellulose, which not only exhibited high thermal stability, but also had lower thermal conductivity than metallic materials [20]. After the chemical treatment and freeze-drying, the lignin and hemicellulose were removed, resulting in a decrease in density from 98 mg/cm^3^ of the natural wood to 31.68 mg/cm^3^ (Figure S2). In addition, the gaseous thermal radiation of wood aerogel-8 h also played a significant role [42]. The multilayer structure of wood aerogel-8 h separated channels for gas transmission, and the large amount of nanopores in the cell walls further restricted the movement of gas molecules, leading to poor gaseous radiation thermal conductivity [12,43]. The low density, multilayer structure, and exposed nanopores reduced the thermal conductivity from 0.113 W/mK of the natural wood to 0.033 W/mK of the wood aerogel-8 h (Figure 7c).

**Figure 8:**
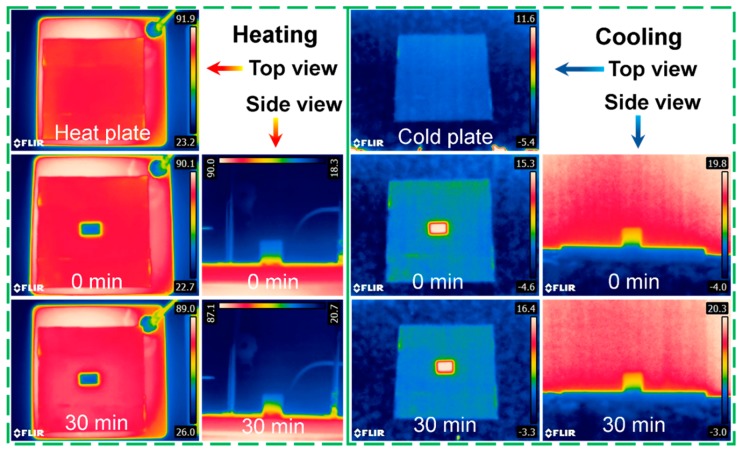
Infrared images of wood aerogel-8 h during the 30 min heating and cooling processes.

To further illustrate thermal insulating properties, the surface temperature variation of wood aerogel-8 h during the heating cooling processes was recorded using an infrared thermal camera as shown in Figure 8. In order to obtain a temperature-stable hot plate, an aluminum plate with a thickness of 1 cm was placed on an electric calefaction plate. The surface of the aluminum plate was covered with a layer of tape (ε = 0.95) to increase the surface emissivity. The wood aerogel-8 h was placed on the ~80 °C hot plate. Due to the low thermal conductivity of the wood aerogel-8 h, the high temperature was observed across the contact surface. After being heated for 30 min, the temperature of the top surface did not change. In the side view, the thermal diffusion along the layer structure was observed. Similarly, the cool plate was obtained by placing the tape-covered aluminum plate on ice. After being cooled for 30 min, the temperature of the top surface did not change and the temperature of the wood aerogel-8 h was close to the environmental temperature.

**Figure 9:**
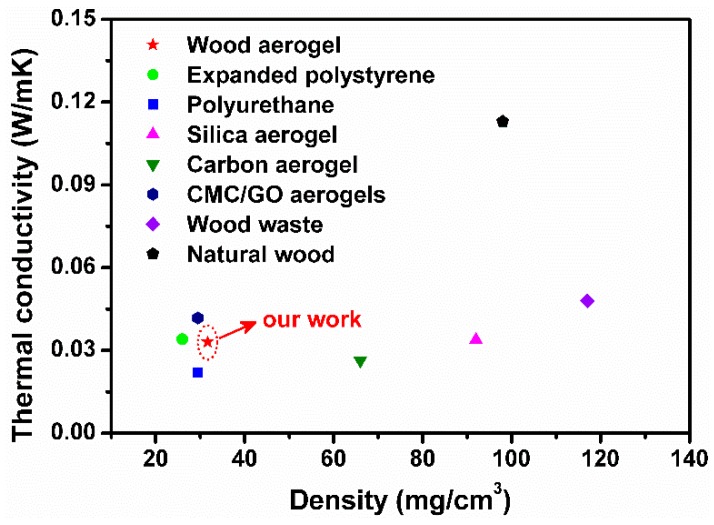
Comparison of the thermal conductivity and density of wood aerogel with reported thermal insulation materials.

In practical applications, a low thermal conductivity was important for thermal insulation materials, while the density of the material was also critical. In Figure 9, the thermal conductivity and density of the wood aerogel-8 h and common thermal insulation materials are compared. Traditional synthetic organic thermal insulation materials such as expanded polystyrene and polyurethane had a low thermal conductivity of 0.022–0.034 W/mK as well as a density of 25–30 mg/cm^3^ [3,44]. However, the drawbacks of releasing toxic gas and poor biodegradability hindered their further applications. Inorganic materials such as carbon aerogel and silica aerogel also exhibited low thermal conductivity of 0.026–0.033 W/mK and their density was slightly higher than organic materials, but they were fragile [8,45]. Wood aerogels directly obtained from natural wood possessed lower thermal conductivity and density than natural wood and wood waste [46]. It is worth mentioning that, although the thermal insulation performance of wood aerogel was lower than traditional organic materials and cellulose-based aerogel [47], the developed wood aerogel in this study showed much better biocompatibility, biodegradability, and mechanical compressibility, as well as simpler process technology [13,48].

## 4. Conclusions

In summary, the lightweight, anisotropic, and mechanical compressible wood aerogel with a multilayer structure was designed and fabricated via a facile method directly from natural wood for application as a thermal insulation material. The removal of lignin and hemicellulose optimized the structure of the natural wood, involving the disappearance of the middle lamella and the degradation of the cellulose amorphous regions and hemicellulose, resulting in low density (~31.68 mg/cm^3^) and high specific surface area (~32.18 m^2^/g). The destroyed ultrathin cell walls and the presence of wood rays gave the wood aerogel a great multilayer structure, endowing wood aerogel with great compressible properties (~33.04 kPa at 60% strain). Moreover, the synergistic effect of the multilayer structure and the micropores reduced the thermal conductivity of the wood aerogel from 0.113 W/mK of the natural wood to 0.033 W/mK of the wood aerogel. The combination of low density, great mechanical compressibility, low thermal conductivity, and biodegradability makes the wood aerogel an ideal candidate for green and cost-effective thermal insulation materials.

## Figures and Tables

**Table 1:** Specific surface area, pore diameter, and pore volume of wood samples.

Sample	BET Specific Surface Area (m^2^/g)	Pore Diameter (nm)	Pore Volume (cm^3^/g)
Natural wood	7.24	12.94	0.019
Delignified wood	17.29	3.81	0.036
Wood aerogel-4 h	18.06	6.69	0.086
Wood aerogel-8 h	32.18	7.63	0.146
Wood aerogel-12 h	21.99	7.55	0.100

## Supplementary Materials

The following are available online at https://www.mdpi.com/2073-4360/12/1/165/s1, Figure S1: Morphology of wood rays, Figure S2: Density of natural wood and wood aerogel.
