# Supplementary material for: Lightweight, Anisotropic, Compressible, and Thermally-Insulating Wood Aerogels with Aligned Cellulose Fibers
Source: Polymers (Basel). 2020 Jan 8;12(1):165. doi: 10.3390/polym12010165 (PMC7022930; doi:10.3390/polym12010165)
Supplement: Supplementary file 1 [file polymers-12-00165-s001.pdf]

Supplementary Materials

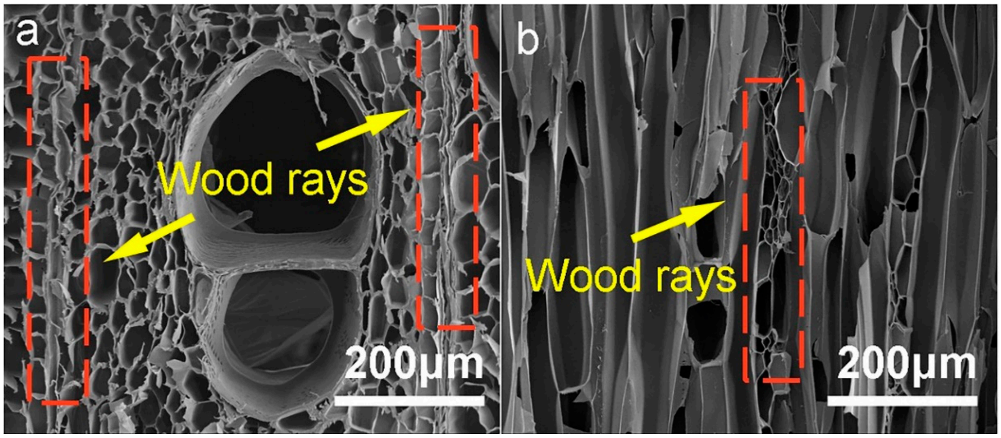

Figure S1. Morphology of wood rays in (a) cross section and (b) tangential section.

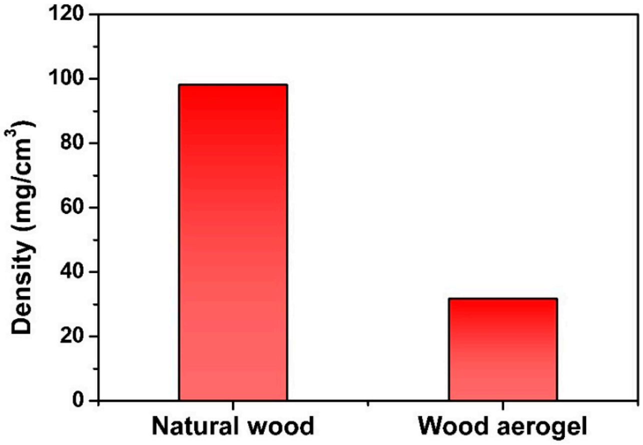

Figure S2. The density of natural wood and wood aerogel.
